# Impact of Exercise on Psychological Well-Being in Patients with Pediatric Cancer: An Experimental Study

**DOI:** 10.3390/children12040404

**Published:** 2025-03-22

**Authors:** Kenan Koç, Tuba Arslan, Osman Pepe, Kerimhan Kaynak, Mustafa Soner Yüce, İbrahim Dalbudak, Özdemir Atar, Berat Koçyiğit, Gül Bahar Bayıroğlu, Şaban Ünver, Hamza Küçük, Levent Ceylan, Fatma Neşe Şahin

**Affiliations:** 1 Faculty of Sport Sciences, Erciyes University, Kayseri 38280, Turkey; kenankoc@erciyes.edu.tr (K.K.); tubaakgul@hotmail.com (T.A.); 2 Faculty of Sport Sciences, Suleyman Demirel University, Isparta 32260, Turkey; osmanpepe@sdu.edu.tr (O.P.); beratkocyigit@sdu.edu.tr (B.K.); gulbayiroglu@sdu.edu.tr (G.B.B.); 3 Faculty of Sport Sciences, Istanbul Sabahattin Zaim University, Istanbul 34303, Turkey; mustafa.yuce@izu.edu.tr; 4 Institute of Health Sciences, Erciyes University, Kayseri 38280, Turkey; 5 Faculty of Sports Sciences, Uşak University, Uşak 64000, Turkey; ibrahim.dalbudak@usak.edu.tr; 6 Faculty of Sports Sciences, Çanakkale 18 Mart Universty, Çanakkale 17100, Turkey; ozdemir@comu.edu.tr; 7 Yaşar Doğu Faculty of Sport Sciences, Ondokuz Mayıs University, Samsun 55270, Turkey; saban.unver@omu.edu.tr (Ş.Ü.); hamza.kucuk@omu.edu.tr (H.K.); 8 Faculty of Sports Sciences, Hitit University, Çorum 19030, Turkey; leventceylan@hitit.edu.tr; 9 Faculty of Sport Sciences, Ankara University, Ankara 06100, Turkey; nesesahin@ankara.edu.tr

**Keywords:** anxiety, pediatrics cancers, depression, exercise, quality of life

## Abstract

**Objective:** This study was conducted to investigate the effects of exercise on psychological disorders in patients receiving childhood cancer treatment. **Methods:** The study group consisted of patients with cancer between the ages of 9 and 17 who were treated in the Hematology–Oncology units of Erciyes University. For the sample group, children with cancer were informed about the content of the study, and 40 children with cancer agreed to participate in the study voluntarily. The volunteers were divided into two groups, control and experimental, each consisting of 20 people. For the pre-test, participants completed a socio-demographic information form, as well as the Kovacs Depression scale, Beck Anxiety Inventory, and the pediatric cancer quality of life scale for children. Volunteers in the experimental group engaged in aerobic and strength exercises for an eight-week period. The study was completed with 8 volunteers in the control group and 14 volunteers in the experimental group due to various factors, such as voluntary withdrawal, disease progression, and mortality. After this period, the volunteers were asked to complete the same scales once more as a post-test. The SPSS 22.00 statistical analysis program was used. The independent samples *t*-test was employed to compare the pre-test and post-test findings of the control and experimental groups, while the paired samples *t*-test was used for within-group evaluations. **Results:** In the within-group comparisons, significant differences were observed in favor of the post-test scores in the experimental group for both the anxiety scale (*p* < 0.05, Mean: 8.14) and the quality-of-life child form (*p* < 0.05, Mean: 38.14). For intergroup comparisons, significant differences were found in favor of the experimental group in terms of post-test scores of depression (*p* < 0.05, mean: 10.57) and anxiety scales (*p* < 0.05, mean: 8.14). **Conclusions:** It is postulated that this outcome stems from the positive effects of sports activities in helping children undergoing cancer treatment distance themselves from their psychological adversities.

## 1. Introduction

Cancer is a disease characterized by the uncontrolled proliferation of cells in the body, leading to damage to the tissue of origin and potentially spreading to surrounding and/or distant tissues [1]. Cancer develops due to environmental and genetic factors. Environmental causes include exposure to radiation and chemicals, viruses, poor nutrition, and smoking, while genetic causes encompass hereditary changes, other genetic factors, hormonal elements, and immune disorders [1]. Cancer is one of the most significant health issues affecting society worldwide. According to World Health Organization data, the global cancer rate increased to 19.3 million new cases and 10.0 million fatalities in 2020. One in every five individuals worldwide will develop cancer during their lifetime [2]. Cancer affects not only adults but also children. Cancer types occurring in the 0–19 age range are referred to as childhood cancers. Childhood cancers constitute 2–4% of all cancers. Annually, at least 400,000 children aged 0–19 are diagnosed with cancer worldwide. In Turkey, this figure is approximately 2800–3000. The top three cancer types seen in children are leukemias at 30%, lymphomas at 20%, and nervous system tumors at 10–15% [3]. Disciplined progression in cancer treatment has led to significant developments, with major advancements achieved in the last 30 years. Survival rates for childhood cancers have now reached 70–80% [4]. There are four main types of treatment commonly used in childhood cancers: chemotherapy, radiotherapy, stem cell transplantation, and surgical treatment [5]. Treatments may be applied individually or in combination, as determined by the physician. After diagnosis and treatment plan determination, a traumatic process and extended treatment period begin for both the family and the child [6]. While cancer is a medical and physical illness, it also encompasses psychological and psychosocial aspects. Children undergoing cancer treatment may exhibit physical symptoms such as vomiting, weight loss, fatigue, and hair and eyebrow loss. In addition to physical symptoms, they also experience psychological symptoms. Cancer resistance can also lead to other mental health problems, such as depression, post-traumatic stress disorder, and phobias [7,8]. The manifestation of the disease can vary depending on the age at diagnosis. In young children, the predominant features are concerns about pain and fear of separation from parents. School-age children begin to experience feelings of loneliness. Adolescents may experience fear of death and stress related to physical changes [9,10]. Based on interviews with two hundred patients, Kübler-Ross et al. [11] described this death-related process in five stages: denial, anger, bargaining, depression, and acceptance. “Patients are often overly grateful to someone who takes a little care of them or gives them a little time. In a world full of devices and numbers, they are so deprived of this kind of attention that they respond extremely well to a little human touch” [11]. Depression and acute anxiety are frequently observed in patients diagnosed with cancer. Symptoms of depression include intense feelings of helplessness and hopelessness. There is generally a pessimistic outlook on life, with the belief that nothing in life can be good and the situation will never improve. Loss of interest in daily activities emerges [12]. Anxiety, also known as anxiety disorder, is characterized by the emergence of anxiety without the presence of danger, its prolonged duration, and its intense experience. These feelings are similar to fear, stress, and worry. The individual may constantly think about the future and feel anxious and concerned about various issues. A person with anxiety disorder may not want to do anything in their daily life because they cannot enjoy activities due to their thoughts. They may have difficulty communicating with family and individuals in their environment, leading to disruption of social life [13]. An individual who cannot communicate may eventually become withdrawn, and the degree of anxiety disorder may increase. Patients diagnosed with cancer should be encouraged to freely express their emotions experienced during these stages and discuss their thoughts about the illness; they should be assisted in improving their quality of life by facilitating psychological and social adaptation and strengthening the relationship between the patient, family, and social interaction areas [14]. It is believed that distancing themselves from play and school environments negatively impacts school performance, fosters a loss of self-confidence, and induces future anxiety, pessimism, and hopelessness when observing other patients undergoing similar treatment, as well as anxious family members in the hospital environment. Meanwhile, peers and friends continue their social lives, while the patient lacks areas for communication or socialization with anyone other than nurses, doctors, and ward staff. They spend days constantly in bed in complete isolation from the external world, which can lead to the loss or reduction in some psychological, social, and physical characteristics of the patient while making medical progress and trigger psychological disorders such as anxiety and depression [15]. The effect of psychological distress on individuals has been a field studied for many years. In many studies in this field, researchers have stated that exercise is beneficial for mental disorders. It has been stated that sports can be beneficial for the psychological and physical aspects of such illnesses [15].

There are many potential benefits of exercise prescription after a cancer diagnosis: improving surgical outcomes, reducing perceived symptoms, managing radiation and chemotherapy-related side effects, maintaining or increasing physical function, improving psychological health, reducing fat gain and muscle–bone loss, and prolonging survival. Therefore, regular exercise should be recommended for the general population, especially for those at a high risk of cancer. Furthermore, exercise should be incorporated into the routine clinical care of patients with cancer as part of treatment to improve quality of life and reduce morbidity and mortality [16,17]. In light of this information, we can observe that physical activity/exercise has positive effects on patients undergoing cancer treatment.

Our review of the literature reveals that exercise studies have been conducted on adult patients with cancer, examining factors such as psychosocial aspects, sleep quality, and quality of life. The researchers conducting these studies are mostly nurses [15,16,17]. There are few studies on childhood cancer and exercise, and again, we observed that the researchers in these studies were nurses. In the literature, it is suggested that nurses conducted physical activity and exercise programs and that having these studies conducted by experts trained in and concerned with the discipline of training and exercise would yield more efficient and positive results. This planned study aims to add original and new information to the literature in light of scientific knowledge and studies, further contributing to science. On the other hand, this research is a very specific study in terms of continuing both online and face-to-face. It is an example of applications that can be performed to support people who are ill remotely during the pandemic period. The objective of this study is to examine and evaluate the effects of exercise on some psychological disorders in children with cancer receiving treatment in the oncology hospital, with the expectation that exercise will have a positive effect both psychologically and physically.

## 2. Materials and Methods

### 2.1. Research Model

In the study, an experimental model was used. The experiments with the highest scientific value were those conducted using true experimental models. Common characteristics of true experimental models are the use of multiple groups and the formation of these groups through random assignment (sampling). Thus, each study includes at least one experimental group and one control group. These are considered “equalized” in terms of other control variables.

Three examples of true experimental models are as follows:

- Pre-test–post-test control group model;
- Post-test control group model;
- Solomon four-group model.

A researcher using a Solomon four-group design must have the resources and time to use four research groups, which is not always possible in research departments with limited funding. Most schools and organizations do not allow researchers to randomly assign four groups because this would disrupt their normal practice [18]. The statistics involved are extremely complex, even in the age of computers and statistical programs. Unless the research is funded by a large budget and a large team of researchers, most experiments are more feasible than pre-test–post-test research designs. For research, the pre-test–post-test model is robust enough and does not require the Solomon four-group design. Pre-test–post-test control group model: In the pre-test–post-test control group model, two groups are formed by random assignment. One of these is used as the experimental group and the other as the control group. Measurements are taken in both groups before and after the experiment [18]. The symbolic view of the model is presented in Table 1.

The presence of pre-tests in the model helps determine the degree of similarity between the groups before the experiment and adjust the post-test results accordingly. In this model, to determine the extent of “X”’s effectiveness, both pre-test and post-test measurement results are used. For this purpose, we observed the following:

- Percentage increases in pre-test–post-test scores for each group are found, and average increases are compared;
- Covariance analysis is performed using pre-test scores as the covariate with post-test scores;
- Pre-test scores (O1.1 and O2.1) are compared, and if there is no significant difference, only post-test scores (O1.2 and O2.2) are used to test the differences between means [18].

### 2.2. Sample Groups

In this study, the study group consisted of patients with cancer who were diagnosed with childhood cancers between the ages of 9 and 17 undergoing treatment at Erciyes University Mustafa Eraslan and Fevzi Mercan Children’s Hospital, Hematology–Oncology unit. These patients had not previously received a psychiatric diagnosis, had not used antidepressants or their derivatives, had been diagnosed with a psychological disorder following their cancer diagnosis, were receiving support from the hospital’s psychological counseling service, had been undergoing cancer treatment, and were receiving outpatient treatment. Volunteer groups were selected using the random sampling method. To create a sample group, children with cancer were informed about the content of the study. A total of 40 volunteer children who were sick, 20 in the experimental group and 20 in the control group, participated in the study. The study initially started with this number of participants. The experimental group was completed with 14 patients due to various factors, such as voluntary withdrawal, disease progression, and mortality. Some patients in the control group chose to withdraw from the study, declining to participate in the final assessment and concluding with 8 participants. The socio-demographic and clinical characteristics of the control and experimental group are shown in Table 2.

### 2.3. Preparation of Exercise Program

When creating the content of the prepared exercise program, the literature was reviewed. Exercise plans previously implemented for patients with cancer were referenced. Aerobic exercise (light or moderate intensity), anaerobic exercise (very light or light), walking, and strength exercises targeting large muscle groups were identified as the main components of the program. Step aerobics, rhythm and dance (light or moderate intensity), educational games, and stretching/relaxation movements, which were thought to be supportive and motivating for pediatric patients, were also included. The duration, frequency, intensity, and type of exercises were adjusted under the guidance of the specialist physician participating in the study by taking into account the medical health conditions of the patients and by taking into account the opinions of the faculty members of the Faculty of Sports Sciences. Before starting the exercise program, the family was informed about the content, process, and possible situations of the study, and a parental consent form was obtained. The exercise program is presented in Table 3.

As seen in Table 3, the experimental group patients undergoing cancer treatment participated in 24 exercise sessions over 8 weeks, with sessions lasting 60 min each, 3 days a week.

### 2.4. The Details of the Exercise Program Are as Follows

1. Warm-up: Neck tilts, shoulder circles, hip circles, chest stretches, torso twists, floor touches, light tempo jumps, and side-to-side lunges.
2. Walk: Walking at a light pace.
3. Stretch: Upper trapezoidal stretching, rhomboid stretching, hip flexor stretching, hamstring stretching, pectoral stretching, piriformis stretching, Quadriceps stretching, gastrocnemius stretching, tensor fascia stretching, and soleus stretching.
4. Cooling down: Full-body lower and upper limb stretches performed slowly. Movements are performed very calmly.

### 2.5. Procedure

**Figure 1:**
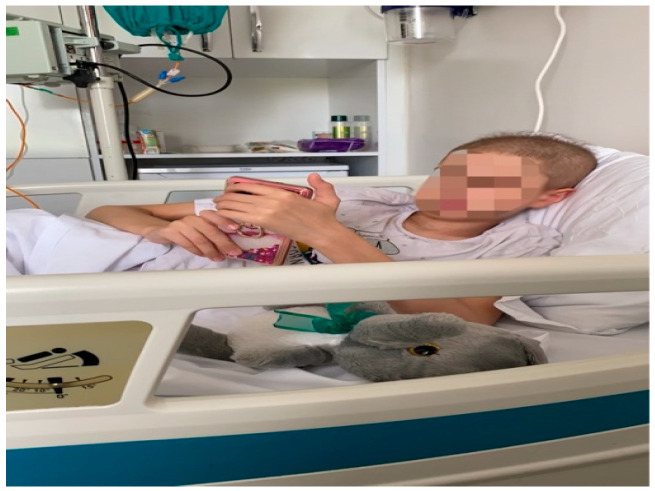
An example image of the control group performing pre-test scales.

As the first step of the study, control and experimental groups were analyzed. The patient’s voluntary consent form, socio-demographic information form, Kovacs depression scale, Beck anxiety scale, and Child form of the pediatric cancer quality of life scale were administered in the presence of the psychologist of the relevant hospital unit. The Health Sciences Ethics Committee with the decision dated 28 July 2021 and numbered 214. The studies were conducted in accordance with the local legislation and institutional requirements. This study was prepared to comply with the Declaration of Helsinki (Figure 1).

In the second step of the study, the exercise program was conducted in the sports halls of Erciyes University’s Faculty of Sports Sciences or in areas suitable for sports that would not create infection and similar problems for the children in the experimental group. Before starting the exercise, the area where sports would be performed was ventilated, and necessary hygiene conditions were ensured. The sessions in the first five weeks of the program were conducted in a face-to-face manner by researchers under the supervision of oncology doctors and psychologists.

**Figure 2:**
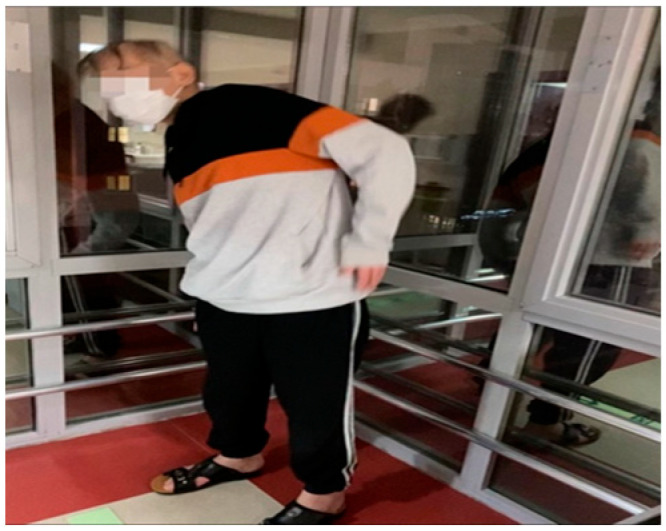
An example image of the experimental group exercising face to face.

To establish standards for comparing parameters to determine the physical conditions of children and exercise programs for the applied exercises, the modified Borg scale, which is generally used in such studies, was used to determine the intensity of the exercise. The Modified Borg Scale (MBS) was developed by Borg in 1970 to measure efforts expended during physical exercise. It is one of the most reliable scales for determining the severity of dyspnea at rest and during exertion, consisting of 10 items describing the severity of dyspnea according to its degree. It is also easier for patients to use and has been shown to correlate with pulmonary function tests. MBS is also superior to other scales in terms of long-term reproducibility. Studies in the literature were examined, and the 2–3 point range, which is considered mild to moderate, was taken as a reference after consultation with oncologists and sports physicians. The scale was taught to the children and placed in a place where they could see it during exercise, and they were asked to report when they felt the severity in the range of 2–3 points, upon which the exercise was interrupted [19,20,21]. In the literature, studies suggest that physical activity and exercise applications were conducted by nurses and that having these studies conducted by experts trained in and concerned with the discipline of training and exercise would yield more efficient and positive results (Figure 2).

**Figure 3:**
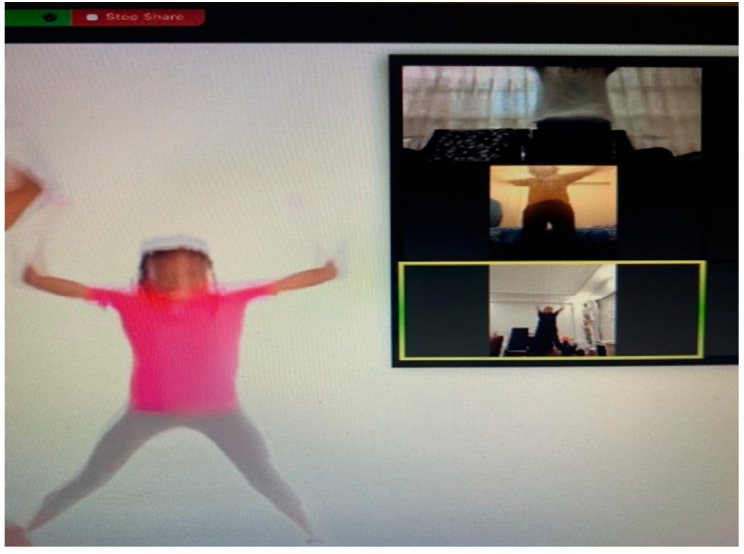
An example image of the experimental group exercising in person and via Zoom.

At the beginning of the sixth week, due to precautions taken in response to the COVID-19 pandemic, the study switched to the Zoom online video platform. Patients in the experimental group participated in video conferences organized by the researcher at consistent intervals. The researcher first demonstrated the exercises and then watched a video replay of the movements. The children then completed the program under supervision by performing the exercises. Patients in the experimental group were viewed from a wide-angle perspective (Figure 3).

**Figure 4:**
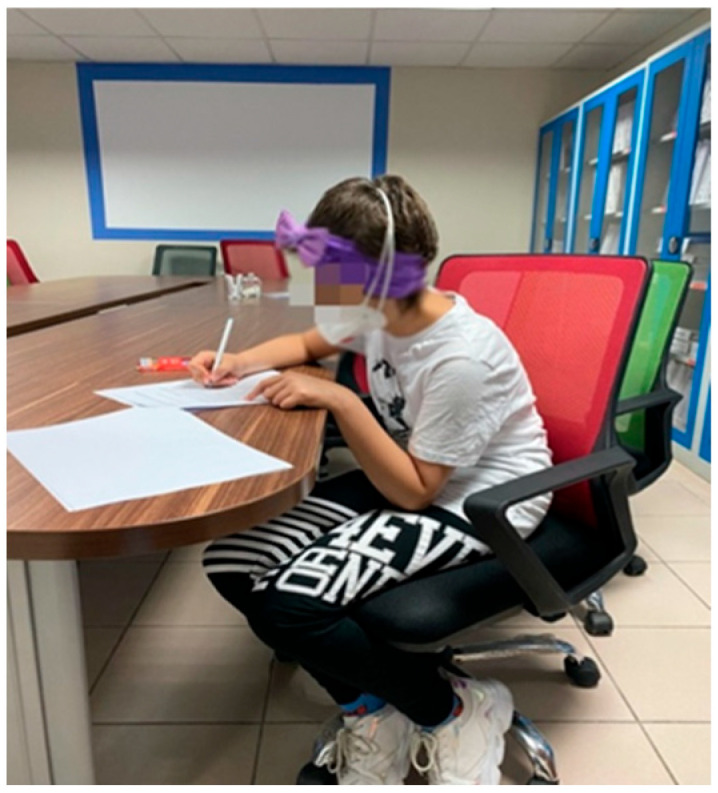
An example image of the experimental group performing post-test scales.

As the third and final step of the study, control and experimental groups were performed. The Kovacs depression scale, Beck anxiety scale, and Child form of the pediatric cancer quality of life scale were administered in the presence of the psychologist of the relevant hospital unit once again (Figure 4).

### 2.6. Data Collection Tools

Volunteers took the socio-demographic information form, Kovacs depression scale, Beck anxiety scale, and Child form of the pediatric cancer quality of life scale. These were administered by the researcher in the presence of a psychologist.

### 2.7. Socio-Demographic Information Form

A form containing 6 questions was prepared by the researchers to obtain personal information such as age, educational status, diagnosis, and duration of the disease.

### 2.8. Kovacs Depression Scale

This scale is a self-assessment tool developed by Kovacs in 1980 [22] based on the Beck depression inventory. This scale, which evaluates the severity of depressive symptoms, is applicable to children and adolescents aged 6–17 years. The scale consists of 26 items, with each item scored 0, 1, or 2 points based on symptom severity. The maximum total score is 54, with a recommended cut-off point of 19. Reverse items in the scale are scored inversely. Higher scores indicate more severe depression [22,23]. The validity and reliability study for Turkish culture was conducted by Öy [23]. Öy [23] reported that the test–retest reliability of the children’s depression scale was reported as 0.70, with an internal consistency of 0.80.

### 2.9. Beck Anxiety Inventory

The Beck anxiety inventory was developed by Beck et al. in 1988 [24]. The scale aims to determine the frequency and severity of anxiety symptoms experienced by individuals. It consists of 21 items, with a maximum possible score of 63. The validity and reliability study for Turkish culture was conducted by Ulusoy et al. in 1998 [25]. They [25] determined the Cronbach’s Alpha internal consistency score of the scale as 0.93.

### 2.10. Pediatric Cancer Quality-of-Life Inventory in 7–18-Year-Old Children

The scale developed by Öztürk in 2008 aligns with the determined characteristics of quality-of-life scales and disease-specific quality-of-life scales found in the literature, specifically the quality-of-life scale for patients with childhood cancer, which consists of 41 items [26]. The scale is a 5-point Likert format. Öztürk [26] reported that Cronbach’s alpha for the child scale is 0.88.

According to Table 4, Cronbach’s Alpha and McDonald’s Omega values obtained from the scales show that the overall scales are sufficiently reliable. The data provided by the participants on the relevant scale show an acceptable level of consistency within themselves.

### 2.11. Data Analysis

Socio-demographic information form, Kovacs depression scale, Beck anxiety inventory, and Child forms of pediatric cancer quality of life were administered, and the results were transferred to the SPSS 22.00 program developed by IBM for necessary calculations. Descriptive scores of volunteer information and scales applied by volunteers were presented as frequency (f) and percentage (%). The normality of data distribution was determined by examining the skewness–kurtosis values. Normality test results of participants are presented in Table 5 and Table 6. Based on this information, it was observed that the data showed a normal distribution and were suitable for parametric tests. Based on this, “Independent Sample T” and “Paired Samples T Test” statistics were used to compare the pre-tests and post-tests of the control and experimental groups.

In Table 5, the results of the normality analysis for the variables were found to be ±1.5. There are some studies in the literature stating that values between ±1.5 [27] and ±2 [28] are acceptable for normal distribution.

## 3. Results

An examination of the pre-test and post-test means and standard deviations of the participants reveals that the experimental group’s depression scores were 12.14 ± 6.47 for the pre-test and 10.57 ± 5.27 for the post-test, with the difference between these scores yielding *t* = 1.348 and *p* = 0.201. For the control group, the depression scores were 15.88 ± 6.62 for the pre-test and 16.75 ± 8.07 for the post-test, with the difference between these scores yielding *t* = −0.628 and *p* = 0.550.

When Table 7 is examined; Analysis of the participants’ pre-test scores revealed that the mean and standard deviation for the experimental group were 12.14 ± 6.47, while for the control group, they were 15.88 ± 6.62. The difference between these scores was found to be *t* = −1.283, *p* = 0.220. Examination of the post-test scores showed that the experimental group scored 10.57 ± 5.27, while the control group scored 16.75 ± 8.07. The difference between these post-test scores was determined to be *t* = −1.942, *p* = 0.041. A significant difference was identified between the post-test scores of the experimental group and the control group. 

When Table 8 is examined; Examination of the participants’ pre-test and post-test means and standard deviations revealed that the experimental group’s anxiety scores were 20.86 ± 5.92 for the pre-test and 8.14 ± 7.92 for the post-test, with the difference between these scores yielding *t* = 7.990, *p* = 0.000. For the control group, the anxiety scores were 27.25 ± 10.46 for the pre-test and 29.38 ± 7.58 for the post-test, with the difference between these scores yielding *t* = −1.042, *p* = 0.332. A significant difference was identified between the pre-test and post-test scores of the experimental group.

When Table 9 is examined; Analysis of the participants’ pre-test scores indicated that the mean and standard deviation for the experimental group were 20.86 ± 5.92, while for the control group, they were 27.25 ± 10.46. The difference between these scores was found to be *t* = −1.590, *p* = 0.080. Examination of the post-test scores showed that the experimental group scored 8.14 ± 7.92, while the control group scored 29.38 ± 7.58. The difference between these post-test scores was determined to be *t* = −6.140, *p* = 0.000. A significant difference was identified between the post-test scores of the experimental group and the control group.

When Table 10 is examined; Analysis of the participants’ pre-test and post-test means, and standard deviations revealed that the experimental group’s quality of life scores were 28.21 ± 8.20 for the pre-test and 38.14 ± 8.24 for the post-test, with the difference between these scores yielding *t* = −3.935, *p* = 0.002. For the control group, the quality-of-life scores were 34.00 ± 5.86 for the pre-test and 34.88 ± 5.82 for the post-test, with the difference between these scores yielding *t* = −0.652, *p* = 0.535. A significant difference was identified between the pre-test and post-test scores of the experimental group.

When Table 11 is examined; Examination of the participants’ pre-test scores indicated that the mean and standard deviation for the experimental group were 28.21 ± 8.20, while for the control group, they were 33.75 ± 6.09. The difference between these scores was found to be *t* = −1.659, *p* = 0.088. When the post-test scores were analyzed, it was calculated that the experimental group had an average of 38.14 ± 8.24, while the control group had an average of 34.88 ± 5.82. Although there was no statistically significant difference, an improvement in the arithmetic mean was found in both the pre- and post-tests.

## 4. Discussion

Childhood cancer treatment involved an 8-week exercise program addressing anxiety, existence, and life. It was determined that the 8-week exercise program applied to patients with childhood cancer positively affects anxiety, depression, and quality of life. As a result of our research, it was determined that patients receiving treatment at the Children’s Hospital had a positive attitude that was above average.

This study implemented a practical program to investigate the effects of moderate exercise on depression, anxiety, and quality of life in patients with pediatric cancer. Regarding the depression variable, the initial pre-test results showed no significant difference between the control and experimental groups. Post-intervention analysis revealed a significant difference between groups, favoring the experimental group. Our literature review yielded the following findings:

Studies demonstrate the positive effects of exercise on individuals. For instance, regular exercise has been shown to reduce tension and effectively decrease depression [29,30,31,32], contribute to feelings of well-being and happiness [33,34], and increase psychological well-being [35]. Malchow et al. [36] found that adolescents engaging in regular physical activity had lower depression scores, while those with minimal or no exercise participation showed significantly higher depression levels. Eime et al. [37] reported fewer depressive symptoms in children who exercised regularly. Janssen and LeBlanc [38] noted that an 8–12-week exercise program positively affected at least one depressive symptom. Rothon et al. [39] observed that each additional hour of weekly exercise reduced depressive symptoms by 8%.

The World Health Organization (WHO) and the UK National Institute for Health and Clinical Excellence (NICE) recommend exercise as a treatment method for depression. Scientific studies have confirmed the antidepressant effect of exercise on both depressive symptoms and diagnosed cases of depression of varying intensities [40,41,42]. Various sources in the literature indicate that regular physical activity and participating in sports prevent the development of certain cancer types [43,44] or reduce the negative effects of cancer [45,46,47,48,49,50,51].

Bar-Or and Rowland [52] evaluated the effect of twice-weekly hospital-based aerobic exercise on 10 post-pubertal cancer survivors who had not received chemotherapy or radiation therapy for at least a year. The results showed positive effects on anxiety, depression, stress management, and quality of life. This study also demonstrates that exercise contributes to reducing depression levels in patients with pediatric cancer. It is thought that physiological and hormonal changes resulting from exercise help alleviate depression by distancing individuals from the negative effects of cancer to some extent.

Regarding the anxiety variable, initial pre-test results showed no significant difference between the control and experimental groups. Post-intervention analysis revealed a significant difference between groups, favoring the experimental group. Our literature review yielded the following findings:

Chang et al. [15] found that patients with acute myeloid leukemia undergoing chemotherapy who participated in a 3-week exercise program (for 12 min daily, at least 5 days a week) experienced less depression and anxiety compared to the control group. Blaauwbroek et al. [53] demonstrated the positive effect of weekly yoga exercises for 7 weeks on anxiety in patients with lymphoma receiving or having received chemotherapy within the last 12 months. Shore and Shepard [54] observed significant improvements in physical fitness and anxiety after a 12-week, 30 min daily aerobic exercise program supervised by an expert and family members. Courneya et al. [16] concluded that a 12-week regular aerobic exercise program for 122 patients with lymphoma in Canada increased the patients’ happiness and reduced their anxiety. These studies align with our research findings. Various exercise movements performed during the intervention help patients with cancer relax, reduce stress, and, as a result, reduce loss.

Regarding the quality-of-life variable, initial pre-test results showed no significant difference between the control and experimental groups. Post-intervention analysis revealed a positive, although not statistically significant, difference between groups, favoring the experimental group. However, a significant difference was observed between the experimental group’s pre- and post-test results, favoring the post-test.

Kürtüncü and Kuğuoğlu [55] reported statistically significant differences in mean quality-of-life scores between groups after a 3-month exercise program for children with acute lymphocytic leukemia. Another study examined the effect of twice-weekly hospital-based aerobic exercise on post-pubertal cancer survivors who had not received treatment or therapy for at least a year [52]. San Juan et al. [56] demonstrated significant improvements in muscle strength, functional mobility, physical fitness, and quality of life in children aged 8–16 who underwent hematopoietic stem cell transplantation and participated in an 8-week aerobic and resistance training program.

Polat et al. [57] and de Almeida and Noll [58] identified a significant relationship between physical activity levels and quality of life. Conversely, Barakou et al. [59] and Luca et al. [60] found that physical activity is one of the most important factors for preventing chronic diseases and improving quality of life.

Some studies have shown that exercise positively affects quality of life, while others have found no significant effect. It is evident that there is no consensus on this matter, and more research is needed. Although our study results were not statistically significant, the positive increase in mean scores may be attributed to people finding relaxation and value in such activities during the pandemic period.

In conclusion, in intragroup comparisons, significant differences were observed in favor of the post-test scores within the experimental group for both the anxiety scale and the quality of life children form. In intergroup comparisons, significant differences were detected in favor of the experimental group regarding the post-test scores of depression and anxiety scales. This phenomenon is attributed to the beneficial impact of physical activity in alleviating the psychological distress experienced by patients with pediatric cancer.

## 5. Recommendations

- Considering this study and the existing literature presented in this study, exercise should be considered a potential supportive element in treatment processes.
- Oncology hospitals should incorporate activities such as music therapy, exercise, and art therapy in addition to primary treatment elements to enhance patients’ motivation and psychological resilience.
- Extensive long-term pilot studies should be conducted with volunteers of different cancer types and age groups.
- Based on the results, relevant authorities should implement additional remedial measures for individuals with poor quality of life, depression, and anxiety conditions.
- Medical school curricula should be expanded to include topics of patient psychology and alternative supportive treatments.
- In order to increase the applicability of such activities and their real-life equivalent, appropriate infrastructures for exercises should be prepared in hospitals or therapy centers.

### Limitations of the Study

- Volunteers who participated in this study died during the process or could not continue because their diseases progressed. This situation disrupted the balance in the numbers of experimental and control group participants at the beginning of the study and made comparisons difficult.
- Some of the research coincided with the COVID-19 pandemic, which necessitated the use of an online platform while implementing the program. Although it did not negatively affect the programs, additional infrastructure services were provided to the volunteers (internet and computers, etc.).
- This study only investigated the psychological benefits of exercise. Future studies examining hormonal effects through blood results may provide more desirable results.

## Figures and Tables

**Table 1:** Symbolic representation of the model.

G1	R	O1.1	X	O1.2
G2	R	O2.1		O2.2

**Table 2:** The socio-demographic and clinical characteristics of groups.

		Control Group		Experimental Group	
	Variable	N	%	N	%
Gender	Male	3	62.5	7	50.0
	Female	5	37.5	7	50.0
Age	12–13	4	50.0	5	35.7
	14–15	1	12.5	6	42.9
	16 and above	3	37.5	3	21.4
Educational Status	Middle School	4	50.0	6	42.9
	High School	4	50.0	8	57.1
Number of Siblings	1	3	37.5	4	28.6
	2	2	25.0	5	35.7
	3 and adove	3	37.5	5	35.7
Disease Diagnosis	Leukemia	2	25.0	7	50.0
	Lymphoma	3	37.5	4	28.6
	Other	3	37.5	3	21.4
Disease Duration	1–180 days	6	75.0	10	71.4
	181 days and more	2	25.0	4	28.6

**Table 3:** An 8-week exercise program repeated in the same way.

	Monday	Wednesday	Friday
3 days a week for 60 min	Warm-up (10 min)	Warm-up (10 min)	Warm-up (10 min)
	Walk (30 min)	Walk (30 min)	Walk (30 min)
	Stretch (10 min)	Stretch (10 min)	Stretch (10 min)
	Cooling down (10 min)	Cooling down (10 min)	Cooling down (10 min)

**Table 4:** Reliability analysis results of the scales.

Scales	Number of Items	Cronbach’s Alpha	McDonald’s Omega
Kovacs Depression	26	0.80	0.79
Beck Anxiety	21	0.93	0.90
Child Form of The Pediatric Cancer Quality of Life	41	0.91	0.88

**Table 5:** Normality test results of participants’ scale scores.

		Control Group		Experimental Group	
Scales	Tests	Skewness	Kurtosis	Skewness	Kurtosis
Kovacs Depression Scale	Pre-test	0.835	0.328	0.835	0.328
	Post-test	0.682	−0.533	0.682	−0.533
Beck Anxiety Inventory	Pre-test	0.135	−1.207	0.135	−1.207
	Post-test	1.257	0.501	1.257	0.501
Child Form of The Pediatric Cancer Quality of Life	Pre-test	−0.232	1.143	−0.232	1.143
	Post-test	−0.044	−1.262	−0.044	−1.262

**Table 6:** Comparison of depression scale pre-test and post-test scores for experimental and control groups.

Scale	Groups	Test	N	M ± Sd	t	*p*
Kovacs Depression	Experimental	Pre-test	14	12.14 ± 6.47	1.348	0.201
		Post-test	14	10.57 ± 5.27		
	Control	Pre-test	8	15.88 ± 6.62	−0.628	0.550
		Post-test	8	16.75 ± 8.07		

Note: From now on, the arithmetic mean will be expressed as M, and the standard deviation will be expressed as Sd.

**Table 7:** Comparison of depression scale scores between experimental and control groups.

Scale	Test	Groups	N	M ± Sd	*t*	*p*
Kovacs Depression	Pre-test	Experimental	14	12.14 ± 6.47	−1.283	0.220
		Control	8	15.88 ± 6.62		
	Post-test	Experimental	14	10.57 ± 5.27	−1.942	0.041 *
		Control	8	16.75 ± 8.07		

* sign is used to indicate that *p* is a significant difference.

**Table 8:** Comparison of anxiety scale pre-test and post-test scores for experimental and control groups.

Scale	Groups	Test	N	M ± Sd	*t*	*p*
Beck Anxiety	Experimental	Pre-test	14	20.86 ± 5.92	7.990	0.000 **
		Post-test	14	8.14 ± 7.92		
	Control	Pre-test	8	27.25 ± 10.46	−1.042	0.332
		Post-test	8	29.38 ± 7.58		

The ** sign is used to indicate that *p* is close to 0.000, that is, a highly significant difference.

**Table 9:** Comparison of anxiety scale scores between experimental and control groups.

Scale	Test	Groups	N	M ± Sd	*t*	*p*
Beck Anxiety	Pre-test	Experimental	14	20.86 ± 5.92	−1.590	0.080
		Control	8	27.25 ± 10.46		
	Post-test	Experimental	14	8.14 ± 7.92	−6.140	0.000 **
		Control	8	29.38 ± 7.58		

The ** sign is used to indicate that *p* is close to 0.000, that is, a highly significant difference.

**Table 10:** Comparison of quality-of-life scale pre-test and post-test scores for experimental and control groups.

Scale	Groups	Test	N	M ± Sd	*t*	*p*
Child Form of The Pediatric Cancer Quality of Life	Experimental	Pre-test	14	28.21 ± 8.20	−3.935	0.002 **
		Post-test	14	38.14 ± 8.24		
	Control	Pre-test	8	34.00 ± 5.86	−0.652	0.535
		Post-test	8	34.88 ± 5.82		

The ** sign is used to indicate that *p* is close to 0.000, that is, a highly significant difference.

**Table 11:** Comparison of quality-of-life scale scores between experimental and control groups.

Scale	Test	Groups	N	M ± Sd	*t*	*p*
Child Form of The Pediatric Cancer Quality of Life	Pre-test	Experimental	14	28.21 ± 8.20	−1.659	0.088
		Control	8	33.75 ± 6.09		
	Post-test	Experimental	14	38.14 ± 8.24	1.085	0.292
		Control	8	34.88 ± 5.82		

## Data Availability

Data are available upon request due to privacy and ethics restrictions.
